# Dimensional crossover in the quantum transport behaviour of the natural topological insulator Aleksite

**DOI:** 10.1038/srep11691

**Published:** 2015-06-29

**Authors:** Pascal Gehring, Kristina Vaklinova, Alexander Hoyer, Hadj M. Benia, Viera Skakalova, Giacomo Argentero, Franz Eder, Jannik C. Meyer, Marko Burghard, Klaus Kern

**Affiliations:** 1 Max-Planck-Institut für Festkörperforschung, Heisenbergstrasse 1, D-70569 Stuttgart, Germany; 2 University of Vienna, Faculty of Physics, Physics of Nanostructured Materials, Boltzmanngasse 5, A-1090 Vienna, Austria; 3 Institut de Physique de la Matière Condensée, École Polytechnique Fédérale de Lausanne, CH-1015 Lausanne, Switzerland

## Abstract

Three-dimensional topological insulators comprise topologically protected surface states displaying massless, Dirac-like linear dispersion with spin-momentum locking. Electrical conduction through such surface states has been documented to manifest itself in a two-dimensional character of the angle-dependent magnetotransport behavior. Here, we explore the size-dependent electronic properties of nanostructures made of the lead-containing mineral Aleksite, a naturally occurring topological insulator. Compared to its sister compound Kawazulite, a member of the well-studied Tetradymite crystal class, the crystal structure of Aleksite is distinguished by its lack of any counterpart within the group of synthetic topological insulators. Low temperature Hall measurements on thin Aleksite nanosheets reveal a significant carrier mobility on the order of 1000 cm^2^/(Vs), and a high carrier density of ***n*** = 3.9 × 10^25^ m^−3^. Importantly, for Aleksite nanoribbons with a width below 150 nm, a 1D weak antilocalization effect along with 1D universal conductance fluctuations emerges, which transforms into 2D behavior for larger ribbon widths

Three-dimensional (3D) topological insulators (TIs) possess a bulk band gap and gapless metallic surface states associated with spin-polarized (helical) Dirac cones^1^. They open up novel aspects in condensed matter physics, and are promising as components of spintronic devices^2^ and for quantum computing applications^3^. However, residual bulk conduction due to defects, as well as thermal excitation across the small bulk band gap, severely limit the exploitation of the surface state features in electronic devices^4^,^5^. Suppression of the bulk contribution has been achieved by enhancing the crystal quality of synthetic TIs^6^, or by the use of TIs with a relatively large band gap such as Bi_2_Se_2_Te^7^. Alternatively, naturally occurring TIs may offer some advantages, including reduced bulk doping as a consequence of defect minimization that occurred over geological time spans. Along these lines, the Tetradymite-type mineral Kawazulite has recently been documented to be a natural TI whose electrical properties are comparable to those of its synthetic counterparts^8^.

According to theoretical studies, minerals belonging to the Aleksite group represent alternative candidates for natural TIs^9^. The prototype mineral Aleksite with the composition PbBi_2_Te_2_S_2_ appears to be particularly promising in this respect for several reasons. Firstly, it has been predicted to possess a relatively large band gap of the order of 0.3 eV^9^. Secondly, it is set apart from most TIs investigated so far, which are closely related to Tetradymite, whose crystal structure (space group 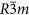) features quintuple layers separated by a van der Waals gap. Aleksite, by contrast, belongs to the space group 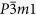 comprising seven-layer blocks that are also separated by van der Waals gaps (see Fig. 1a). Thirdly, it enables addressing the general issue whether or not the presence of Pb and S may induce a phase transition from a TI to a trivial insulator^10^.

## Results

### Chemical and structural analysis

**Figure 1:**
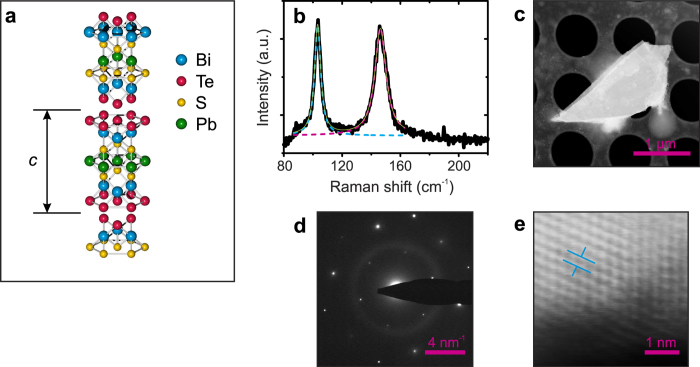
Spectroscopic and microscopic analysis of Aleksite. (**a**) Schematic depiction of the layered structure of Aleksite, with seven atomic layers constituting a covalently bonded block. These blocks are held together by van der Waals interaction. Bismuth, tellurium, sulphur and lead atoms are shown in blue, red, yellow, and green, respectively. (**b**) Raman spectrum of an Aleksite microcrystal, acquired under ambient using a laser wavelength of 633 nm. (**c**) Transmission electron micrograph of a mechanically exfoliated Aleksite sheet on a holey carbon grid. (**d**) Transmission electron diffraction pattern obtained from the sheet in panel (**c**). (**e**) Transmission electron image (4.4 × 4.4 nm^2^) taken close to the edge of the sheet. The [1-100] spacing, marked by the two lines, is ~0.35 nm.

The Aleksite crystals investigated in this work were purchased from Mikon Mineralienkontor (Klein Lengden, Germany) and originate from the Kochkar district in southern Urals, Russia. By energy-dispersive X-ray spectroscopy (EDX), their detailed composition was determined to be Pb_1_Bi_2.06_Te_1.79_S_1.54_ (see Supplementary Information). Individual microcrystals were investigated by confocal Raman spectroscopy. Figure 1b depicts the low-wavenumber region of a typical Raman spectrum recorded using a red laser with *λ* = 633 nm. The two prominent peaks at 103.2 cm^−1^ and 146.3 cm^−1^ can be identified as the E_2g_ and the A_21g_ peaks common for layered chalcogenides, respectively^11^. The upshift of the A_21g_ peak compared to Bi_2_Te_3_ (A_21g_ = 134 cm^−1^)^12^ arises from the replacement of heavy Te by the lighter S in Aleksite.

Exfoliated Aleksite flakes (see Fig. 1c) were subjected to electron diffraction analysis and direct imaging with atomic resolution. Selected-area diffraction patterns (Fig. 1d) were recorded within the central area of the flake, showing a single crystal structure with hexagonal projected symmetry (the [1–100] spacing corresponds to 0.351 nm). High-resolution annular-dark field STEM imaging was carried out at the edges of the flake, since the central regions were too thick. Around the edges, a poly-crystalline structure with multiple orientations of the crystal was found, as exemplified in Fig. 1e. Lattice spacings of 1.34, 1.72, 1.83, 2.76, and 3.01 Å were observed for different orientations of the crystallites, in reasonable agreement with the spacings previously found by X-ray diffraction^13^ and with calculated data^9^.

### Spectroscopic characterization

**Figure 2:**
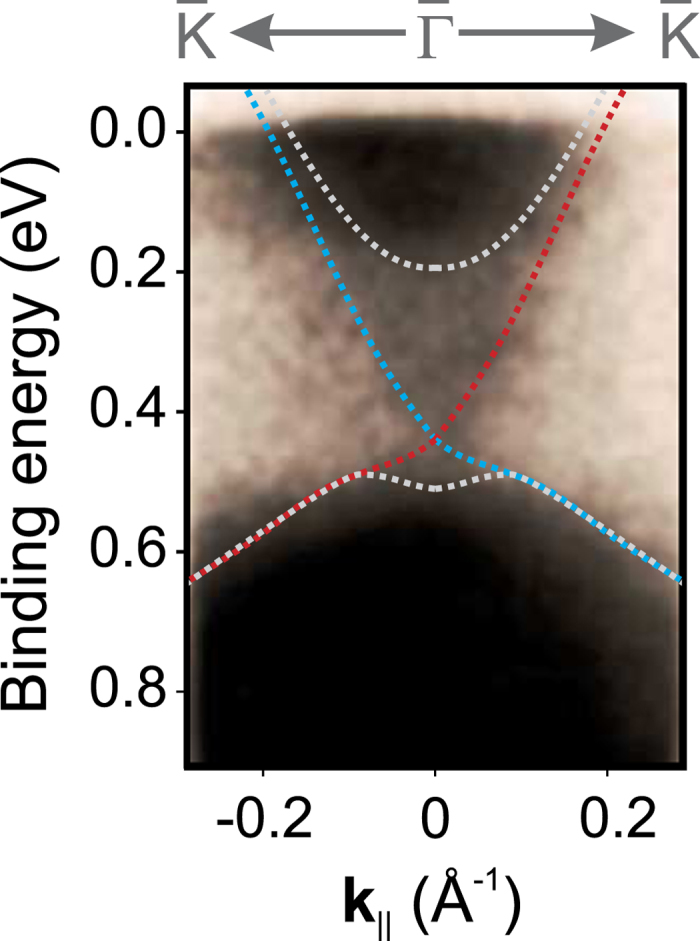
Photoemission spectrum of Aleksite. Experimental surface band structure of an Aleksite microcrystal recorded at 100 K. For visual comparison, the calculated projected bulk band structure of Aleksite along the 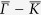
direction, adapted from ref^9^, has been overlaid in the form of dotted lines of different colours (white for the bulk band edges, and blue and red to highlight the topological surface state). The bottom horizontal axis displays the parallel component of the wave vector for the experimental spectrum, while the top horizontal axis belongs to the calculated band structure.

An indication that Aleksite constitutes a natural TI could be gained by angle-resolved photoemission spectroscopy (ARPES) on a tiny single crystalline domain of the mineral. After cleaving the Aleksite crystal in air, it was directly transferred to the UHV chamber and cooled to 100 K. Owing to the very small sample size, only a blurred experimental surface band structure could be recorded (Fig. 2). Nonetheless, the presence of a cone-like shaped band inside the band gap, typical for topological surface states, is supported by direct comparison with the theoretically calculated, projected bulk band structure^9^. The experimental data point toward a doping-induced shift of the Fermi level by more than 0.4 eV above the Dirac point, *i.e.*, well within the bulk conduction band. Similar strong n-type doping has been experimentally observed for PbBi_4_Te_7_^14^. From the apparent edges of the projected conduction band (binding energy at ~0.19 eV) and valence band (binding energy at ~0.5 eV) in the ARPES spectrum, the bulk band gap is estimated to be of the order of 300 meV. This larger value in comparison to the gaps reported for PbBi_2_Te_4_ and PbBi_4_Te_7_ (both below 200 meV)^15^ can be attributed to the substitution of heavy Te atoms by lighter S, which is expected to increase the size of the bulk band gap^9^.

### Magnetotransport behaviour

**Figure 3:**
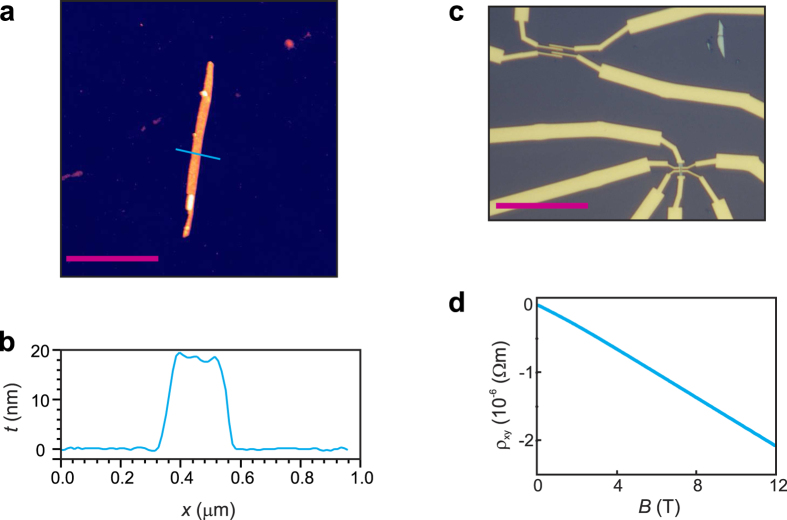
Electrical characterization of Aleksite. (**a**) AFM image of an Aleksite nanoribbon, which randomly formed during mechanical exfoliation (the corresponding electrical transport data are shown in Figs 4 and 5). (**b**) AFM height profile of the ribbon along the blue line in panel (a). (**c**) Optical image of the ribbon contacted with electrode fingers (top), and a thin flake contacted in Hall geometry (bottom). (**d**) Hall resistivity as a function of the external magnetic field, recorded at 1.4 K. Scale bars: (a) 2 μm, (c) 20 μm.

In order to explore the electrical properties of Aleksite, we prepared thin nanosheets and -ribbons via micromechanical cleavage of the microcrystals. In Fig. 3a, a ~20 nm thick (see Fig. 3b) and ~130 nm wide nanoribbon randomly formed during exfoliation is shown. We also observed thin platelets with a thickness as small as 10 nm. Individual thin nanostructures were identified by their optical contrast, followed by standard e-beam lithography to define electrical contacts, and finally thermal evaporation of contact metals (4 nm Ti / 60 nm Au). To ensure Ohmic contacts, the contact regions were pre-treated with Ar plasma for 50 s. Figure 3c depicts a typical device in “finger-like” (top panel) and Hall bar (bottom panel) geometry. Carrier density and mobility were determined by DC Hall measurements at 1.4 K. A typical Hall curve is depicted in Fig. 3d. Linear fits to the data of six individual devices yielded a carrier density *n* in the range of 2.2–4.8 × 10^25^ m^−3^. This finding is in agreement with the ARPES results, thus confirming that the Fermi level is located within the bulk conduction band. Moreover, Hall mobilities between 810 and 2006 cm^2^/(Vs) were extracted from the data, which is slightly higher than reported for Kawazulite^8^.

**Figure 4:**
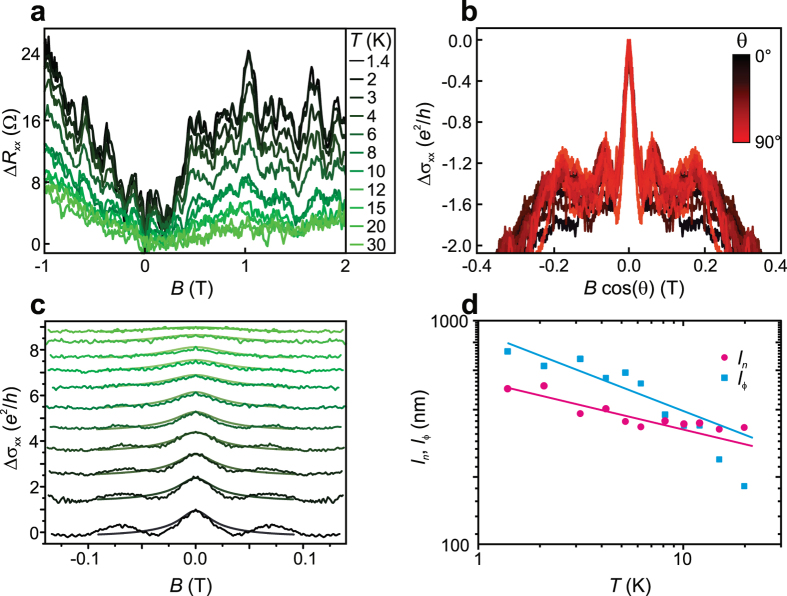
Weak anti-localization in Aleksite. (**a**) Corrected magnetoresistance of the ~20 nm thick, ~140 nm wide and 1.6 μm long ribbon in Fig. 3a for different temperatures. (**b**) Corrected magnetoconductance as a function of the *B*-field component *B* cos(*θ*) normal to the sample surface. (**c**) Fits of the temperature dependent magnetoconductance (colours are identical to those in panel (a)) using equation (2). (d) Nyquist (pink) and zero-field phase coherence length (blue) extracted from the fits in panel (c) as a function of *T*. The solid lines are fits to the data, specifically 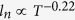 and 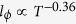.

The very low four-terminal resistance of thin, 5–10 μm large Aleksite flakes on the order of several Ohms resulted in low resistance changes of only several 100 mΩ at the highest external magnetic field (*B* = 12 T). Consequently, the magnetotransport behaviour of such type of samples could not be evaluated. Nonetheless, this task could be achieved using thin Aleksite ribbons, which exhibit a larger resistance of several 100 Ω. The corrected low-field magnetoresistance Δ*R*_*xx*_(*B*) = *R*_*xx*_(*B*) −  *R*_*xx*_(*B*  = 0) of the ~140 nm wide ribbon in Fig. 3a is depicted in Fig. 4a for a range of different temperatures. Around zero *B*-field, a pronounced weak anti-localization (WAL) resistance minimum can be observed.

## Discussion

In order to investigate the dimensionality of the WAL, the sample was tilted in the external magnetic field. It can be seen from Fig. 4b that the WAL only depends on the *B*-field component 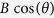 normal to the sample surface, and hence originates from a transport channel with a dimensionality lower than three. On that basis, we have tested data fits using the 2D^16^ Hikami–Larkin–Nagaoka formula Δσ(*B*) = *α*(*e*^2^/*h*)[In(*B*_*ϕ*_/B) − Ψ(1/2 + *B*_*ϕ*_/B)], where *h* is Planck’s constant, *B*_*ϕ*_ is the dephasing field, α is a parameter related to the number of conducting 2D channels, and Ψ(*x*) is the digamma function. The fit of the data in Fig. 4a yields a phase coherence length of *l*_*ϕ*_ ≈ 700 nm from the relation 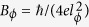, and values between −3 and −5 for the parameter *α* (see Table 1). From the latter range one would have to conclude the presence of an unrealistically large number of conducting channels^17^. A hint toward a lower than 2D character of the ribbon arises if one assumes an extended 2D system with two decoupled and parallel transport channels by fixing *α* = −1^18^. In this case, still the same phase coherence length of *l*_*ϕ*_ ≈ 700 nm is obtained, which is considerably larger than the width *W* *=* 140 nm of the ribbon. The 1D character is further consolidated by the fact that the WAL correction is on the order of *e*^2^/*h*, much higher than typically observed for 2D systems^19^. Accordingly, we attribute the WAL to 1D diffusive transport in the device.

To analyse the magnetotransport data within a 1D framework, we use a localization model developed by Altshuler *et al.*^20^,^21^ for the conductance correction *δ*σ:


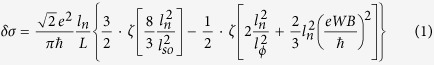


where *L* is the distance between the inner voltage probes, *ζ* 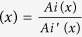 , with *Ai* and *Ai’* as the Airy Ai function and its first derivative, respectively, *l*_*n*_ is the dephasing length governed by low-energy Nyquist scattering, *l*_*ϕ*_ is the zero-field phase coherence length associated with phase-breaking mechanisms different from the Nyquist one, and *l*_*so*_ is the spin–orbit length. This model can be slightly modified by calculating Δσ(*B*) = *δσ*(*B*) − *δσ*(*B* = 0):


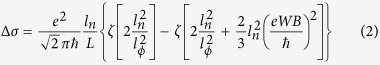


which is independent of *l*_*so*_. The corresponding fits of the magnetoconductivity at different temperatures are presented in Fig. 4c. Their excellent quality underscores the validity of the 1D model for the present samples. The fits yield a Nyquist length of *l*_*n*_ = 496 nm and a phase coherence length of *l*_*ϕ*_ = 728 nm at *T* = 1.4 K. Analysis using different sizes of the fitting window (90 mT, 120 mT and 170 mT) yielded similar values. Furthermore, using the conductance correction *δ*σ at *B* = 0 T in connection with equation (1), a spin–orbit length *l*_*so*_ = 70 nm is estimated. The low spin–orbit length reflects strong spin–orbit interaction in Aleksite, in analogy to other TIs like Bi_2_Se_3_^1^.

**Figure 5:**
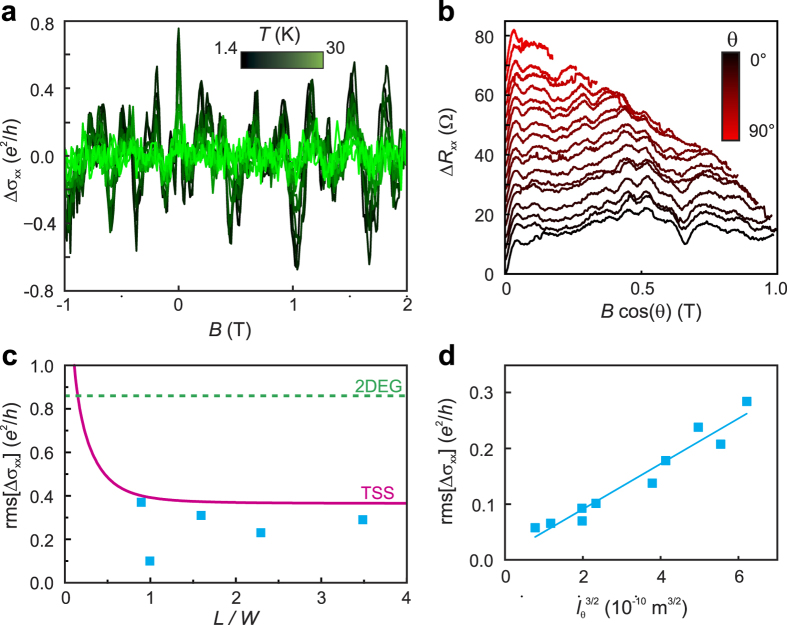
Universal conductance fluctuations in Aleksite. (**a**) UCF amplitude obtained by subtracting a smoothed background from the magnetoconductance for different temperatures. (**b**) Angle-dependent UCFs as a function of the out-of-plane component of the magnetic field. (**c**) Comparison between the amplitude of the measured UCFs (blue) and the calculated values for a topological surface state (pink) and a trivial two-dimensional electron gas (green). (**d**) Root-mean-square of the oscillations as a function of the zero-field phase coherence length obtained from the 1D fits to the WAL data (see Fig. 4d).

From the temperature dependence of the two characteristic lengths in Fig. 4d, it is apparent that both decrease with increasing temperature. Curve fits revealed a 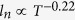 dependence for the Nyquist length, and a 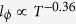 dependence for the phase coherence length. According to theory, the dephasing length should decay with *T*^−1/2^ if the system is 2D and *T*^−1/3^ if it is 1D^22^. The fact that both fit exponents are reasonably close to −1/3 further consolidates the 1D nature of the observed WAL. The diffusive charge transport in the Aleksite ribbons is accompanied by pronounced universal conductance fluctuations (UCF), as clearly discernible in Fig. 4a. In Fig. 5a, the corresponding data are plotted after subtraction of a smoothened background. The amplitude of the oscillations is approximately 0.3*e*^2^/*h*. For 1D systems where *W* < *l*_*ϕ*,*tot*_ < *L* the UCF amplitude should be determined by the ratio of the total phase coherence length 
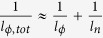
 and the length *L* of the system, *i.e.*, 
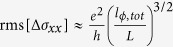
23. For the ribbon under investigation, *L* = 500 nm and *l*_*ϕ*,*tot*_ = 295 nm at 1.4 K (see Table 1), such that rms[Δ*σ*_*xx*_] should be 0.45*e*^2^/*h* which is close to the value of 0.3*e*^2^/*h* observed in our experiments. This agreement again underlines the 1D character of the ribbons.

To investigate the dimensionality of the observed UCFs, we studied their angle dependence. Figure 5b depicts the magnetoresistance data for different tilting angles as a function of the out-of-plane component of the magnetic field. It is evident that most features remain at the same value of 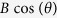 and thus show only little dependence on the *B*-field component *B*_*||*_ parallel to the sample surface. As closed trajectories on the sample surface do not contain flux induced by *B*_*||*_^24^, this observation points against the bulk as origin of the UCFs, and hence supports their low dimensional character. To further distinguish between UCFs related to a topological surface state (TSS) and a trivial, two-dimensional electron gas (2DEG), we follow a strategy suggested by Li *et al.*^24^. Theory predicts an UCF amplitude of 
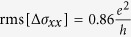
 for a normal 2DEG, whereas


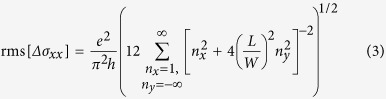


for a TTS. Figure 5c compares our experimental data (blue squares) with a curve obtained using equation (3) (pink line). The former fall only slightly below the values expected for a TTS, but on the contrary are significantly smaller than the values expected for a trivial 2DEG (indicated by the green dashed line). This finding strongly favours topological surface states underlying the UCFs.

With increasing temperature, the UCF amplitude decreases as a result of the reduced phase coherence length. In general, the root–mean–square of the oscillations and the phase coherence length are related by 
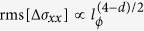
, where *d* is the dimensionality of the system^25^. Correspondingly, for a 1D system 
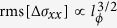
, which is well confirmed by the plot in Fig. 5d (a linear fit to rms[Δ*σ*_*xx*_] vs. *l*_*ϕ*_ failed), where the phase coherence lengths were taken from Fig. 4d.

The results of further magnetotransport experiments on Aleksite ribbons of larger width are summarized in Table 1. Remarkably, for ribbons wider than 300 nm the magnetotransport data could be smoothly fitted using the 2D HLN model, which provided the expected α ≈ −1. Moreover, the resulting phase coherence lengths were on the order of or smaller than the ribbon width *W*, and rms[Δ*σ*_*xx*_] was found to scale linearly with the phase coherence length in wider ribbons as expected for 2D behaviour. All the above observations taken together provide evidence for a cross–over from 2D to 1D diffusive transport when the ribbon width falls below approximately the total dephasing length *l*_*ϕ,tot*_ which should be dominated by the smaller value of *l*_*ϕ*_ and *l*_*n*_.

In summary, we have gained evidence that the mineral Aleksite is a naturally occurring TI that introduces an additional type of crystal structure within this rapidly expanding class of materials. Despite its pronounced deviation from non-stoichiometric composition, Aleksite displays electrical transport performance comparable to the thoroughly investigated synthetic TIs like the Tetradymite-type bismuth chalcogenides. The magnetotransport properties of Aleksite ribbons in dependence of width clearly signify a cross-over from 1D to 2D behaviour. However, it should be emphasized that this observation alone is no direct proof for the contribution of topological surface states to the electrical transport. Nonetheless, the existence of a low-dimensional transport channel with relatively large coherence makes Aleksite a very interesting material for future studies.

## Methods

### Electrical measurements

For electrical characterization, the samples were placed in a pumped bath cryostat (Oxford Instruments) at 1.4 K. AC measurements were performed using two lock-in amplifiers (Signal Recovery 7265). The DC Hall experiments were carried out using an ADwin Gold card combined with a voltage amplifier (Stanford Research SR560) and a current amplifier (Femto DLPCA-200).

### TEM characterization

With the aid of a Scotch tape, a thin layer was exfoliated from a bulk Aleksite crystal, deposited onto a Si substrate covered with 300 nm of SiO_2_, and then transferred on a TEM grid. Electron diffraction was studied by a Philips CM200 Transmission Electron Microscope using electron energy of 80 kV. Scanning TEM (STEM) imaging was carried out with a Nion UltraSTEM 100 operated at 60 kV.

### ARPES investigation

A small (less than 0.5 × 0.5 mm^2^), thin (~0.25 mm) piece of Aleksite was set-apart from a bigger mineral piece using a scalpel. The piece was then glued using electrically conductive glue on the sample holder. Immediately before introduction into UHV, the sample surface was exfoliated with a Scotch tape. The ARPES measurements were performed with a hemispherical SPECS HSA3500 electron analyzer characterized by an energy resolution of about 10 meV. Monochromatized He I (21.2 eV) radiation was used as photon source. During measurements the sample was cooled with the aid of liquid nitrogen to 100 K.

## Additional Information

**How to cite this article**: Gehring, P. *et al.* Dimensional crossover in the quantum transport behaviour of the natural topological insulator Aleksite. *Sci. Rep.* **5**, 11691; doi: 10.1038/srep11691 (2015).

## Supplementary Material

Supplementary Information

## Figures and Tables

**Table 1:**
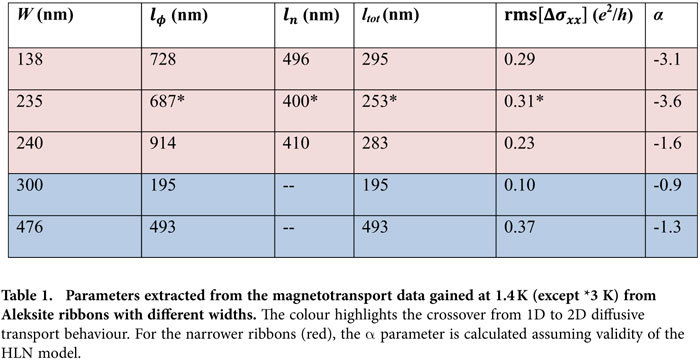
Parameters extracted from the magnetotransport data gained at 1.4 K (except *3 K) from Aleksite ribbons with different widths.
