# Supplementary material for: Dimensional crossover in the quantum transport behaviour of the natural topological insulator Aleksite
Source: Sci Rep. 2015 Jun 29;5:11691. doi: 10.1038/srep11691 (PMC4483779; doi:10.1038/srep11691)
Supplement: Supplementary Information [file srep11691-s1.pdf]

Supplementary Information

Dimensional crossover in the quantum transport behaviour of the natural topological insulator Aleksite

Pascal Gehring[1,4,*], Kristina Vaklinova[1], Alexander Hoyer[1], Hadj M. Benia[1], Viera Skakalova[2], Giacomo Argentero[2], Franz Eder[2], Jannik C. Meyer[2], Marko Burghard[1] & Klaus Kern[1,3]

[1]*Max-Planck-Institut für Festkörperforschung, Heisenbergstrasse 1, D-70569 Stuttgart, Germany*

[2]*University of Vienna, Faculty of Physics, Physics of Nanostructured Materials, Boltzmanngasse 5, A-1090 Vienna, Austria*

[3]*Institut de Physique de la Matière Condensée, Ecole Polytechnique de Lausanne, CH-1015 Lausanne, Switzerland*

[4]*Present address: Department of Materials, University of Oxford, 16 Parks Road, Oxford OX1 3PH, United Kindom*

[*]*pascal.gehring@materials.ox.ac.uk*

The EDX spectrum of a freshly cleaved Aleksite crystal is shown in Figure S1. In addition to electron microscopy analysis, we tried to further investigate the Aleksite by single crystal X–ray diffraction (SC-XRD) and powder XRD measurements. However, owing to the small specimen size below 200 µm, we were unable to fit the obtained data by the Rietveld method, and hence could not determine the lattice constants *a* and *c*.

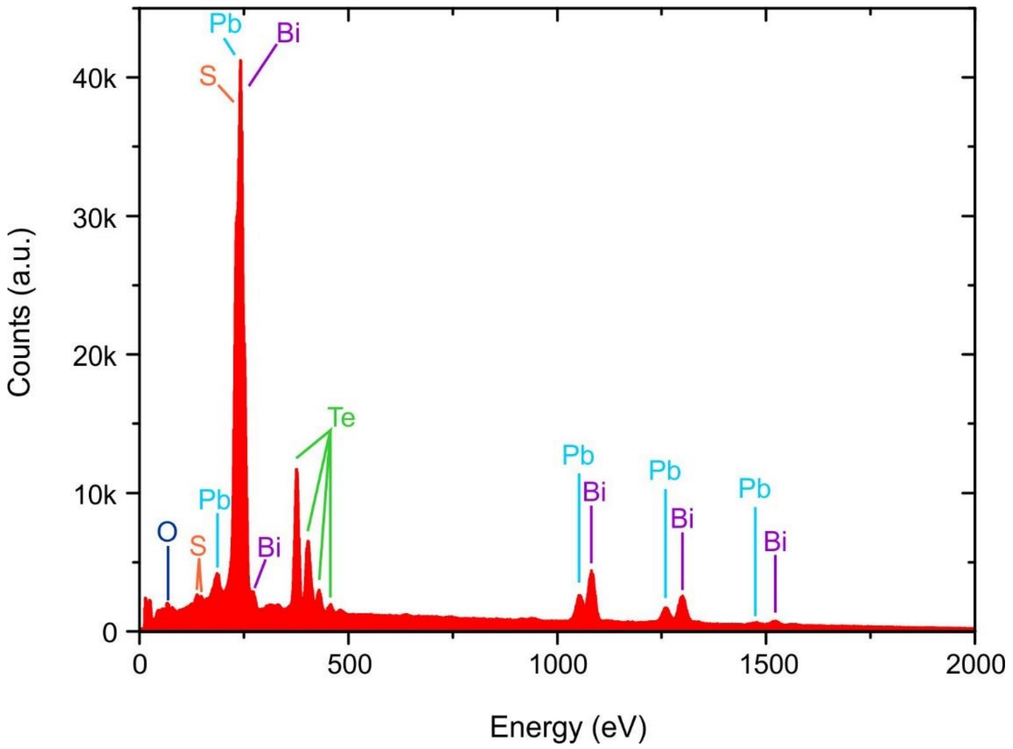

Figure S1: Energy-dispersive X-ray spectroscopy. Taking into account only the elements Bi, Pb, Te and S yields an average stoichiometry of $Pb_1Bi_{2.06}Te_{1.79}S_{1.54}$.
